# Simultaneous reconstruction of septic composite defects in lower extremities: Combination of fasciocutaneous perforator flap and Masquelet technique

**DOI:** 10.3389/fsurg.2022.900796

**Published:** 2022-08-24

**Authors:** Xuanzhe Liu, Jin Yang, Hongshu Wang, Shengdi Lu, Cunyi Fan, Gen Wen

**Affiliations:** ^1^ Shanghai Jiao Tong University affiliated Sixth People’s Hospital; ^2^ First Affiliated Hospital of Kunming Medical University

**Keywords:** fasciocutaneous flap, perforator flap, masquelet technique, composite defects, infection

## Abstract

**Background:**

Management of composite defects with deep infection is a challenge to reconstructive surgeons. This study aimed to demonstrate the versatility, safety, and complications of simultaneous reconstruction of infectious composite defects with fasciocutaneous perforator flap combined with the Masquelet technique.

**Methods:**

This study presents 10 patients in whom a fasciocutaneous perforator flap combined with the Masquelet technique was used to restore soft tissue and bone defects of the lower extremity, and were admitted in two level 1 trauma centers in Shanghai. The first stage included debridement of necrotic bone and infected tissues, implantation of a polymethylmethacrylate cement spacer to cover the void; bridging fixation of the osseous defect using external or internal fixators, and soft-tissue reconstruction with a fasciocutaneous perforator flap. The second stage included cement spacer removal with membrane preservation, refreshing bone edges, and grafting the cavity with bone morphogenetic proteins and autologous iliac bone graft.

**Results:**

The mean follow-up duration after autologous bone graft was 17.5 months. The average bony defects and average flap dimensions were 7.1 cm and 44.9 cm^2^, respectively. All flaps survived uneventfully. No recurrence of infection was detected in either the second stage of surgery or follow-up period. The mean duration of bone consolidation was 31.9 weeks. One patient had a 2 cm leg length discrepancy, and one patient had mild foot drop. No residual deformity requiring a secondary procedure occurred.

**Conclusion:**

Fasciocutaneous perforator flap combined with Masquelet technique provides a reliable and versatile alternative for patients with composite defects resulting from lower extremity infection.

## Introduction

Most cases of composite tissue defects with deep infection result from severe open fracture, which impair the surrounding soft tissue envelope and the blood supply to the bone. The initial injury may compromise the healing potential as well as the response of the host's defense mechanisms against contaminating microorganisms. The treatment of infectious composite tissue defects presents a challenging problem to orthopedics and plastic surgeons.

The main tasks during reconstruction of composite tissue defects with deep infection include adequate bone and soft tissue coverage, elimination of dead space, and reconstruction of the soft tissue envelope with good blood supply to prevent recurrence of infection.

The induced membrane technique (IMT), or Masquelet technique, is increasingly used to address pseudoarthroses and bony defects (1). Studies have revealed that the addition of antibiotics to the cement spacer is effective in promoting the viability of the induced membrane (IM) (2). Shah et al. also reported that antibiotics contained in the spacer had positive effects as it reduces bacteria inoculation and promotes higher osteogenic gene expression (3).

A neurocutaneous flap based on the sural nerve has been widely used for reconstruction of the lower extremity since Masquelet introduced this technique in 1992 (4). As its main advantages, these distally based cutaneous flaps enable preservation of major arterial axes of the extremities and ensures abundant blood supply to the flap.

In this study, we present a series of the combination of two “Masquelet techniques” in a one stage procedure to treat composite tissue defects with deep infection in the lower extremities. We also evaluated its safety and efficiency during the follow-up period. Thus, this study aimed to demonstrate the versatility, safety, and complications of simultaneous reconstruction of infectious composite defects with fasciocutaneous perforator flap and Masquelet technique.

## Patients and methods

The study was approved by the Ethics Committee of Shanghai Jiao Tong University Affiliated Sixth People's Hospital. Informed consent was obtained from all patients. All study methods were conducted in accordance with the principles of the Declaration of Helsinki.

### Patients

Between January 2018 and November 2019, 10 patients who sustained composite tissue defects in the lower extremities caused by deep infections were treated by the combined use of IMT (Masquelet technique) and neurocutaneous flap in two level-one trauma centers in Shanghai as shown in Table 1. All of patients present septic composite defects in lower extremities were included in this study.

**Table 1:** The demographic and injury characteristics of this series.

No.	Gender	Age	Etiology*	Location of defects	Area of defects (length × width)	Length of bone defects	Bacterial pathogen
1	M	45	RTA	distal third of lower extremity (fibular side)	9 cm × 5 cm	8.5 cm	*Staphylococcus aureus*
2	M	51	RTA	distal third of lower extremity (tibia side)	6 cm × 4 cm	7.5 cm	*Pseudomonas aeruginosa*
3	F	55	RTA	ankle (tibia side)	6.5 cm × 5 cm	6 cm	*Staphylococcus aureus*
4	M	32	Crush injury	distal third of lower extremity (tibia side)	8.5 cm × 5.5 cm	5.5 cm	*Staphylococcus aureus*
5	F	49	RTA	ankle (fibular side)	7 cm × 4.5 cm	7 cm	*Staphylococcus aureus*
6	M	55	RTA	ankle (tibia side)	7.5 cm × 6 cm	3.6 cm	*Pseudomonas aeruginosa*
7	M	33	RTA	middle third of lower extremity (anterior side)	8 cm × 6.5 cm	6.5 cm	*Staphylococcus aureus*
8	M	42	RTA	ankle (tibia side)	10 cm × 6 cm	9 cm	*Escherichia coli*
9	M	38	Crush injury	ankle (fibular side)	10.5 cm × 6 cm	8.5 cm	*Staphylococcus aureus*
10	M	60	RTA	distal third of lower extremity (tibia side)	9 cm × 5.5 cm	9 cm	*Staphylococcus aureus*

* RTA, road traffic accident.

### Data collection and evaluation

Preoperative data of all patients were collected through a review of their charts. Preoperative information included smoking history, diabetes history, movement function, and sensation of the affected limb. The patients were evaluated at 3, 12, 24, and 36 months after undergoing reconstructive surgery, or after frame removal in patients who wore frames. At each point, patients were evaluated by an orthopedic surgeon and a physical therapist to ascertain their limb status and the presence or absence of major complications. Major complications were defined as the existence of one of the following conditions: partial or complete flap necrosis that required additional surgery, recurrent infection that could not be controlled using sensitive antibiotics and debridement, refracture without a second injury, hardware failure, recurrent osteomyelitis, donor-site morbidity, and residual deformities that required additional surgery.

### Surgical technique

#### First stage

The first stage included debridement of bone and soft tissues, irrigation, implantation of a cement spacer, bone stabilization using external fixator, and soft tissue reconstruction with a fasciocutaneous perforator flap. Both a peroneal artery perforator sural neurocutaneous flap and a posterior tibial artery perforator saphenous neurocutaneous flap were used in this series. Free flap that could cover critical soft-tissue defect was not included in this study.

The three most common pathogenic bacteria identified were *Staphylococcus aureus*, *Pseudomonas aeruginosa*, and *Escherichia coli*. Therefore, we used 2 g of vancomycin or gentamicin per 40 g of cement prepared.

The color and temperature of the flaps were routinely monitored postoperatively. Anticoagulant and antispasmodic medications were administered intravenously for 7 days postoperatively.

#### Second stage

In this series, the time interval between the two stages was 4–6 weeks, which is also recommended by other studies (5). The second stage included removal of the cement, second debridement, refreshment of both bone ends, and implantation with autologous bone grafts (ABGs) and bone morphogenetic proteins (BMPs); an allograft was added to the ABGs if needed. The bone graft conditions were documented in Table 2 as well. The IM was incised longitudinally to enable replacement of the cement spacer with ABGs, and the remaining part of the IM was carefully protected from being stripped during the manipulation.

**Table 2:** Treatment and follow-up data.

No	Type of flap^a^	Type of bone grafts	Type of fixation	Timing of second stage (weeks)	Timing of surgery from initial trauma (months)	Complication of flap	Complication of bone consolidation	Other complications	Time from 2nd stage to total non-protect weight bearing (weeks)
1	PAP-SN	ABGs + BMPs + AG	Ex/ring	4	0.5	None	None	None	36
2	PTAP-SaN	ABGs + BMPs	Ex/monolateral	4	2	None	None	Mild dropping foot	34
3	PTAP-SaN	ABGs + BMPs	Ex/ring	5	3	None	None	None	32
4	PTAP-SaN	ABGs + BMPs	Ex/monolateral	4	4	None	None	None	31
5	PAP-SN	ABGs + BMPs	Ex/ring	5	2	None	None	None	29
6	PTAP-SaN	ABGs + BMPs+AGs	Ex/ring	5	3	None	None	None	28
7	PTAP-SaN	ABGs + BMPs	Ex/monolateral	6	1	None	None	None	30
8	PTAP-SaN	ABGs + BMPs + AG	Ex/ring	4	3	None	None	None	32
9	PAP-SN	ABGs + BMPs	Ex/ring	4	2	None	2 cm LLD	None	33
10	PTAP-SaN	ABGs + BMPs + AG	Ex/monolateral	4	5	None	None	Superficial pin-site infection	34

^a^ PAP-SN, peroneal artery perforator sural neurocutaneous flap; PTAP-SaN, posterior tibial artery perforator saphenous neurocutaneous flap; ABGs, autologous bone grafts; BMPs, bone morphogenetic proteins; AG, allograft; LLD, leg length discrepancy.

## Results

The demographic and injury characteristics of the 10 patients in this study are presented in Table 1. The major etiology was road traffic accident. The most common bacterial pathogen was *S. aureus*. The mean length of bone defects after the first debridement was 7.1 cm ± 1.8 cm.

### Clinical results

Weeks and months after initial trauma patients received the combined surgery was averaged of 2.5 ± 1.3 months as in Table 2. The mean follow-up period was 17.5 months. No recurrence of infection was noted in all 10 patients. The mean time from the second stage of surgery to total non-protected weight bearing was 31.9 weeks. The mean consolidation from the second stage to total non-protected weight bearing time per centimeter was 4.5 weeks/cm.

For the neurocutaneous perforator flap, all flaps survived uneventfully, and the flap donor site was closed primarily with a skin graft. One patient had a 2 cm leg length discrepancy and one patient had mild foot drop; both patients were treated conservatively. No residual deformity that required a secondary procedure was noted.

### Case example

**Figure 1:**
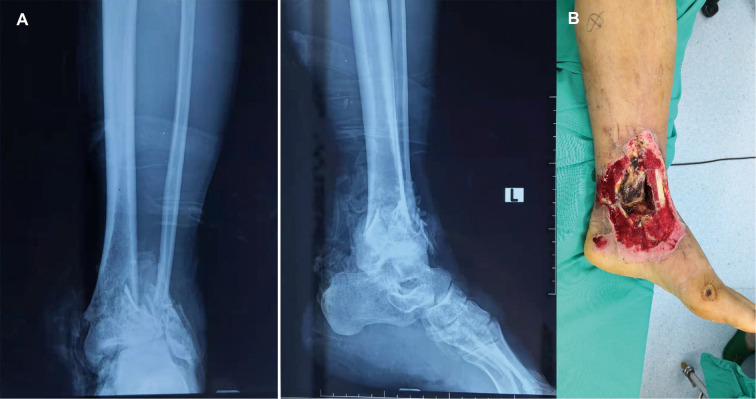
(**A**) A 55-year-old man sustained injury due to a road traffic accident that resulted in a Gustilo grade IIIB fracture in the left medial ankle. Anteroposterior and lateral X-ray radiographs show a tibial and fibula bone fracture. (**B**) Soft tissue defects in the medial ankle and distal tibia shows sign of infection and necrosis.

**Figure 2:**
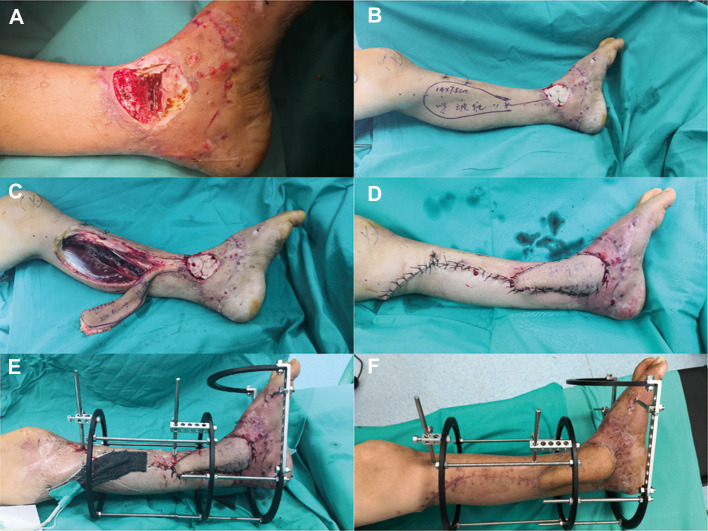
(**A**) Granulation tissue and a 3.6-cm bone defect after thorough debridement and VSD. (**B**) In the first stage, the tibial and talar bone defects were filled with antibiotic-loaded bone cement. (**C**) A distally based saphenous neurocutaneous perforator flap (size, 14 × 7.5 cm^2^) was designed on the patient's left calf. (**D**) Soft tissue defects were covered appropriately. (**E**) The bone was stabilized with an external fixator that bridged the ankle joint. (**F**) The flap totally survived for 5 weeks after the first stage.

**Figure 3:**
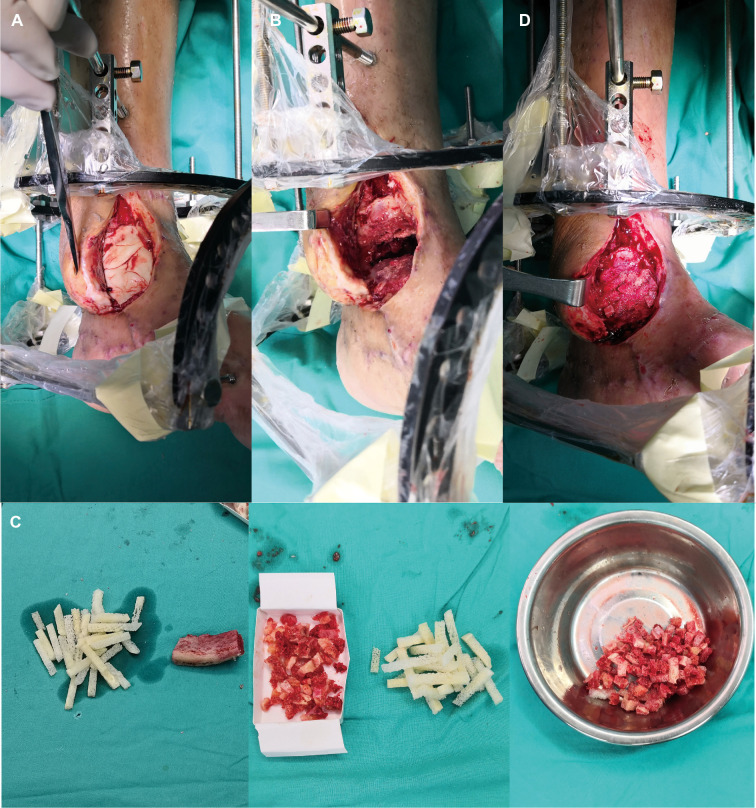
(**A**) The flap was lifted partially and IM was cut. (**B**) The bone cement was then removed. (**C**) The iliac crest was harvested as ABGs. (**D**) The defect was filled with ABGs, AGs and BMPs.

**Figure 4:**
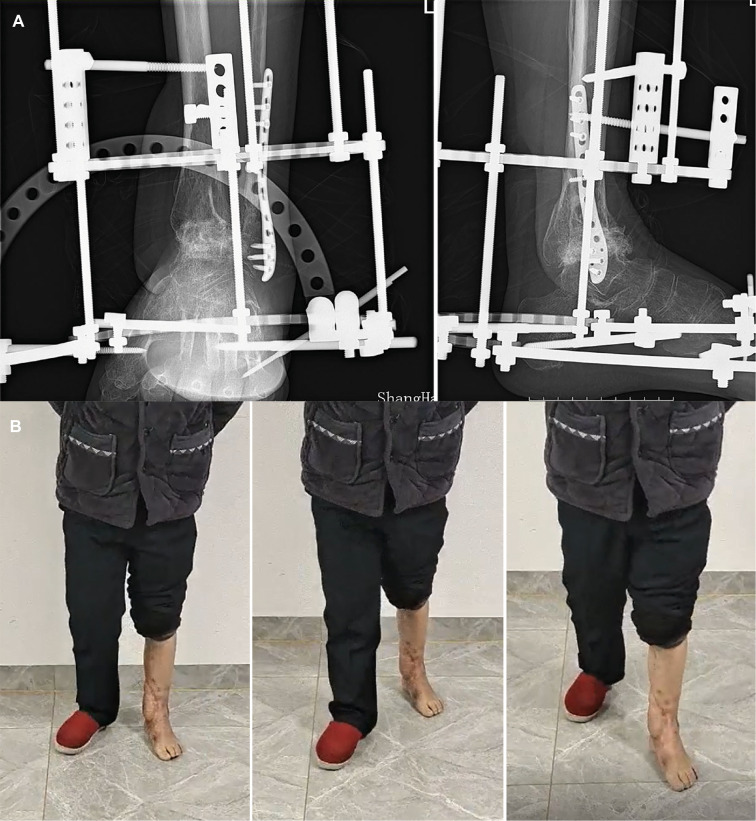
(**A**) The radiograph shows fracture healing 16 weeks postoperatively. (**B**) Appearance and gait at the 22-month follow-up.

Figure 1 depicts a 55-year-old male patient who sustained a traffic injury, resulting in a Gustilo grade IIIB fracture in the left medial ankle. Debridement and ample irrigation with solution were performed in the emergency department of a local hospital. The wound was covered by vacuum sealing drainage (VSD), and the distal tibia and fibula meta-epiphyseal fractures were stabilized with Kirchner wires. As the wound and distal tibia revealed sign of infection and necrosis in the following month, the patient underwent several debridements. The wound extended and the articular surface was exposed. He was then transferred to our trauma center in Shanghai. A swab culture of the purulent wound grew *P. aeruginosa* that was resistant to cefepime and gentamicin. Antibiotic treatment with piperacillin-tazobactam (4.5 g given intravenously every 8 h) was commenced. The soft tissue defects shown in Figure 1B indicated signs of infection and necrosis. All nonviable tissues were thoroughly debrided. All Kirchner wires and necrotic bones were removed, resulting in a 3.6 cm bone defect, and the wound was covered with VSD. Within 5 days, internal fixation of the fibula fracture with a plate and debridement of the necrotic distal tibia and articular surface of talus were performed as in Figure 2A. During the first stage of bone defect management, the tibial bone defect was filled with antibiotic-loaded bone cement (Figure 2B) and stabilized with an external fixator that bridged the ankle joint (Figure 2E). A distally based saphenous neurocutaneous perforator flap was transferred to repair the wound (size, 14 cm × 7.5 cm) (Figures 2C,D). The flap totally survived for 5 weeks after first stage as Figure 2F. The second stage was performed 5 weeks later, the bone cement was removed (Figures 3A,B), and the void was filled with ABGs, AGs, and BMPs (Figures 3C,D). At 6 months postoperatively, the graft appeared completely integrated. The patient was satisfied with the outcome and reported no pain during walking over the following 14 months (Figures 4A,B).

## Discussion

Composite tissue defects with deep infection continue to be one of the most intractable challenges for orthopedic and plastic surgeons. The reconstruction procedures should not only provide reliable soft tissue coverage and bone length maintenance but should also potentially prevent recurrent infections.

The IMT, or Masquelet technique, was first described in 1980 s and was employed to manage patients with infection at the site of fracture nonunion (1, 6, 7). However, no researcher, even Masquelet himself, has mentioned observing the presence of an IM at that time (8). The critical role of the IM was established by two important studies. First, the sheep model study demonstrated that the membrane without bone graft was inefficient and that bone graft without a membrane was rapidly resorbed (9). Thereafter, the *in vitro* study demonstrated that the membrane was a synovium-like epithelium (highly vascularized) that was responsible for the secretion of osteoinductive factors (10).

The IM was later proved to be an organized pseudosynovial rich-vascularized membrane that developed following a foreign-body inflammatory reaction (10). The role of an IM in bone reconstruction and enhancement of tissue healing ablity have also been confirmed by several animal experiments (11–16). Commonly, the delay between initial trauma and the combined surgery indicated the long course of disease and poor tissue healing ablities of patients. In our studies, time between initial trauma and combined surgery averaged of 2.5 ± 1.3 months. IM could provide a benign tissue healing environment with well blood supply. Thus, no eventful complications were documented in our studies. However, the procedures, promotion, and suppression factors of IM formation remain unclear. Several groups have made modifications to the first stage of the surgery in an effort to improve outcomes or uncover mechanisms. Most of these studies have focused on altering the space itself. This is understandable given the large form of the previous foreign-body membrane work, which indicates that implant surface characteristics (e.g. material, surface topography, and hydrophobicity) influence cell adhesion and behavior, thus ultimately changing membrane formation. On the contrary, few studies have focused on factors that influence IM formation from the extrinsic environment of the IM.

The idea of using a neurocutaneous perforator flap for soft tissue reconstruction was derived from our hypothesis that foreign-body inflammatory reaction may benefit from enhancing the blood supply to the surrounding soft tissue envelope. Prior to IMT, Masquelet reported the use of flaps of nutrient vessels of the cutaneous nerve of the lower limb (4). Sural or saphenous neurocutaneous perforator flaps based on the sural artery or tibialis posterior artery have a reliable blood supply with reduced donor site morbidity, achieved by sparing the source artery and underlying muscle and avoiding a microsurgical procedure (17). The main advantage of the neurocutaneous perforator flap is that a well-vascularized adiponeurofascia formed by the vascular plexus of the cutaneous nerve can provide a viable vascularized environment that not only supports soft tissue healing but also prevents infection recurrence (17, 18). Simmilarly, P. G. di Summa et al. has also reported that the radial collateral propellor perforator flap of lateral arm flap has a shorter operation time, hospitalization and higher aesthetic score than the reverse-flow flap (19). Practically, neurocutaneous perforator flap has a similarity with propeller flap as they both have perforators. However, the neurocutaneous perforator flap has a larger blood irrigation field due to the existing traffic branches of “true anastomosis” between the perforator and the initiated neurovascular plexus than propeller flap (20). In terms of the twisted pedicles, the propellor flap has to be freed a longer pedicule to enable the twist of the flap when compared with the perforator flap. Besides, the long vascular pedicule of propellor flap exposed to the underlying cement spacer might be irritated by the inflammation response of the foreign body, thus might easy to cause vasosapam and failure of the operation.

Other than propellor flap, combined technique of free flap and masquelet technique has been revealed as an excellent technique to treat the critical sized soft tissue and bone defect(>8 cm) (21). Surely, it is undeniable that the free flap has an irreplaceable advantage of wide applicability of nearly all soft tissue and the stability of donor blood vessels. Nevertheless, given operation time and the necrosis probability of lifting flap during the second stage osteosynthesis period, the neurocutaneous perforator flap has more advantages as shorter operation time and less flap necrosis rate. Furthermore, if taken sensory recovery into consideration, the results were still debatable. The neurocutaneous perforator flap might also have a better outcome according to our previous study (22). Meantime, sensory recovery of ALT was also proven to have a nice result (23). In Masquelet's latest review, he pointed that appropriate soft tissue reconstruction techniques can give rise to a healthy and well-perfused soft tissue envelope in the defect area (24). Hence, we believed that the choose of neurocutaneous perforator flap is still open to question.

The results of these series demonstrated satisfactory outcomes in either stage 2 bone formation or infection control. Planned consolidation and non-recurrence of infection were noted in all 10 patients during the follow-up period. Our results highlight the potential benefits of neurocutaneous perforator flaps in IM formation and infection prevention. The principal reason of no recurrence of infection was attributed to the vascularized induced membrane by cement spacer. As previous studies reported, the metalwork was a risk factor for the propeller flap (PPF) in soft tissue reconstruction (25). The failure of PPF and the underlying metalwork was believed to the poor blood supply of local soft-tissue bed, as the metal work interfere the original blood perfusion. The similar cause of infection is also caused by the lack of blood supply to the tissue (26). In our cases, benefit from the induced membrane, local blood perfusion was enhanced and thus the potential of infection rate decreased.

There are several studies on the relationship between second stage timing and bone consolidation. Wang et al., reported that the levels of growth factors released were comparable between weeks 4 and 6 after cement spacer insertion (27). Aho et al., observed high levels of vascular endothelial growth factor and interleukin-6 on human IM biopsies, but the osteogenic capacity was reduced in cultures older than 4 weeks (28). In this series, the thickness of the IM were not significantly different between different timings of the second stage (4–6 weeks) under gross observation. Data on the levels of growth factors and more markers of bone marrow-derived mesenchymal cells in each week after spacer implantation are needed to confirm the possibility of early IM maturation in case of soft tissue reconstruction by neurocutaneous perforator flap.

The major limitation of this series was the relatively small sample size. Another limitation in our study is that Masquelet technique was chosen according to the surgeon's experiences. Further prospective comparative studies with other existing methods of managing septic composite defects are required. Our further works include a prospective comparative study on the types of soft tissue reconstruction (i.e., free flap. muscle flap, perforator flap, and neurocutaneous flap) combined with the Masquelet technique in treating septic composite defects and an animal study to evaluate factors from the extrinsic environment of the IM.

The combination of the two Masquelet techniques is reliable and feasible for the reconstruction of septic composite defects in the lower extremities and has a low incidence of complications. This technique is effective for avoiding recurrence of infections and maintaining limb length, although staged procedures are required. Clarity on the details of the technique itself and the mechanism behind it should be further investigated.

## Data Availability

The raw data supporting the conclusions of this article will be made available by the authors, without undue reservation.
